# The Respiratory Exchange Ratio is Associated with Fitness Indicators Both in Trained and Untrained Men: A Possible Application for People with Reduced Exercise Tolerance

**DOI:** 10.4137/ccrpm.s449

**Published:** 2008-02-01

**Authors:** Arnulfo Ramos-Jiménez, Rosa P. Hernández-Torres, Patricia V. Torres-Durán, Jaime Romero-Gonzalez, Dieter Mascher, Carlos Posadas-Romero, Marco A. Juárez-Oropeza

**Affiliations:** 1 Department of Basic Science, Biomedical Science Institute, UACJ, Cd. Juarez Chih, Mexico; 2 School of Physical Education and Sport Sciences, UACH, Chihuahua, Chih., Mexico; 3 Department of Biochemistry, School of Medicine, National Autonomous University of Mexico, D.F., 04510, Mexico; 4 Chemistry School, University of Guanajuato, Guanajuato, Gto., Mexico; 5 Department of Physiology, School of Medicine, National Autonomous University of Mexico, D.F., 04510, Mexico; 6 Department of Endocrinology, National Institute of Cardiology, I.CH., D.F., Mexico

**Keywords:** anaerobic threshold, physiological steady state, oxidative metabolism

## Abstract

**Background:**

The respiratory exchange ratio (RER) indirectly shows the muscle’s oxidative capacity to get energy. Sedentarism, exercise and physically active lifestyles modify it. For that reason, this study evaluates the associations between RER during sub-maximum exercise and other well established fitness indicators (body fat, maximum heart rate, maximum O_2_ uptake, workload, and lactate threshold), in physically active trained and untrained men.

**Methods:**

The RER, O_2_ uptake and blood lactate were measured in eight endurance trained and eight untrained men (age, 22.9 ± 4.5 vs. 21.9 ± 2.8 years; body mass, 67.1 ± 5.4 vs. 72.2 ± 7.7 kg; body fat, 10.6 ± 2.4% vs. 16.6 ± 3.8% and maximum O_2_ uptake, 68.9 ± 6.3 vs. 51.6 ± 5.8 ml•kg^−1^•min^−1^), during maximum exercise test and during three different sub-maximum exercises at fixed workload: below, within or above the lactate threshold.

**Results:**

Endurance trained men presented higher O_2_ uptake, lower blood lactate concentrations and lower RER values than those in untrained men at the three similar relative workloads. Even though with these differences in RER, a strong association (p < 0.05) of RER during sub-maximum exercise with the other well established fitness indicators was observed, and both maximum O_2_ uptake and lactate threshold determined more than 57% of its variance (p < 0.05).

**Conclusions:**

These data demonstrate that RER measurement under sub-maximum exercise conditions was well correlated with other established physical fitness indicators, despite training condition. Furthermore, the results suggest that RER could help obtain an easy approach of fitness status under low exercise intensity and could be utilized in subjects with reduced exercise tolerance.

## Background

The Respiratory Exchange Ratio (RER) (CO_2_ production/O_2_ uptake) increase with the exercise intensity and measured under steady state conditions is commonly used to indirectly determine the relative contribution of carbohydrate and lipids to overall energy expenditure (Simonson and DeFronzo, 1990; Pendergast et al. 2000). A high RER indicates that carbohydrates are being predominantly used, whereas a low RER suggests lipid oxidation (Simonson and DeFronzo, 1990; Pendergast et al. 2000).

The physical fitness indicators used under submaximal exercise tests, like heart rate, lactate and ventilatory thresholds (Achten and Jeukendrup, 2003; Billat et al. 2003), as well as predictive submaximal exercise tests (Noonan and Dean, 2000) have been sufficiently studied and validated; however, the RER, which indirectly shows the muscle oxidative capacity to get energy, has not been validated for this purpose.

Sedentary lifestyle increases the RER values, but decreases the insulin sensitivity, muscle oxidative capacity and contributes to decrease whole body fat oxidation (Morio and Hocquette, 2001; Smorawiński et al. 2001; Rimbert et al. 2004). For that reason, physical inactivity could promote increases in body fat. On the contrary, physically active and trained subjects exhibit lower RER than untrained subjects in response to comparable workloads (Jeukendrup et al. 1997; Bergman and Brooks, 1999). Also, endurance training decreases the RER values, increases the oxidative enzyme activity, O_2_ uptake and delays the time necessary to reach fatigue status during exercise (Messonnier et al. 2005). In addition, a single bout of aerobic and resistance exercise has been shown to decrease the RER, at least for the next 24 h post-exercise (Jamurtas et al. 2004). However, despite the above findings, the exercise effect on the RER changes has not been quantified. These data suggest that controlling factors affecting RER such as diet and previous exercise, the RER values could be properly considered as an auxiliary physical fitness indicator.

The maximal exercise test is the gold standard to assess the aerobic fitness in healthy subjects, including patient with disabilities (Campbell et al. 2004); however, it is not always the most appropriate approach, neither an evidence of daily physical activity levels (Vanhees et al. 2005; D’Alonzo et al. 2006). This limiting of maximal exercise test is mainly applied in subjects with reduced exercise tolerance (neophyte exercisers, sedentary individuals, or those with either pulmonary disease, coronary artery disease, peripheral arterial disease or musculoskeletal pain) (Vanhees et al. 2005; D’Alonzo et al. 2006).The RER at the end of one maximal exercise has been shown a significant correlation with changes in exercise capacity (Stratmann, 1991), and during exercise could be also utilized to evaluate metabolic responses in subjects with reduced exercise tolerance and in physical handicap subjects. To provide additional experimental evidences regarding possible associations between the RER and physical fitness variables, and also to assess the RER under submaximal conditions as a parameter of physical fitness, we evaluated body fat, maximum O_2_ uptake (VO_2max_), lactate threshold (LT), as well as the RER under three different submaximal exercises at fixed workload (SEFW), in trained and untrained physically healthy active men.

## Methods

### Participants

Sixteen healthy male subjects volunteered for the study. Relevant anthropometric characteristics of them are presented in Table 1. Eight subjects were athletes trained at a competitive level (6 tri-athletes and 2 cyclists) and eight were untrained subjects, not enrolled in any exercise program; however, they were all physically active at their worksite. Additional inclusion criteria for trained subjects were: body fat <20%, no body mass changes during the previous 6 months, cardio-respiratory endurance training for more than 6 h per week during, at least, the year preceding the study. For untrained subjects additional inclusion criteria were: no physical training in the preceding year and less than 8 h per month of participation in recreational physical activities. Each participant signed a written informed consent, and the study protocol was approved by the Ethics Committee of the Autonomous University of Chihuahua (Mexico).

### Experimental design

The evaluations were done between 08:00 and 11:00 h in 6 sessions, after 10–12 h overnight fasting and 8–9 h of sleep. Subjects were further instructed to refrain from any sort of heavy physical labour study. The tests were performed in a room with environmental temperatures kept between 22 and 25 ºC. At the first session anthropometrical assessments, diet studies and a practice session on bicycle ergometer were performed. On the second and third sessions, the subjects completed a maximum exercise test during which heart rate, workload (in watts), O_2_ uptake (VO_2_) and CO_2_ production (VCO_2_) were continuously recorded and blood lactate was assayed (see below). From fourth to sixth session, subjects performed at random three different SEFW, during which the described parameters were recorded.

### Anthropometric measures

Two expert anthropometrists, using an anthropometric kit (Rosscraft Tom Kit, Canada) and following the International Society of Advancement in Kinanthropometry (ISAK) technique as described by Norton and Olds (Norton and Olds, 1996), performed the measurements and assessed body fat. Precision and reliability measurements for skin folds, diameters, and body girths measurements were: percentage of technical error 6.2, 1.5, 1.7, and interclass correlation coefficient 0.98, 0.99, 0.99, respectively. Data were analyzed with LifeSize software, version 2.0 (Nolds Sports Scientific; Australia).

### Diet study

The food intake was assessed for three sequential days (one during the weekend) by the 24-hour dietary record method. All dietary records were checked out through interviews. We asked the subjects to keep their diet habits during the study. The dietary records were analyzed with the Diet Balancer software, version 1.4c (Nutridata Software Co, NY). This computer software has a section for Mexican foods, and the defaulting foods were added using food tables from National Institute of Medical Science and Nutrition, Salvador Zubirán, Mexico (Marvan et al. 2006).

### Metabolic measures

In order to determine VO_2_, and RER, the percentages of O_2_, CO_2_ in inspired and expired air as well as minute pulmonary ventilation were measured with a gas analyzer (Sensor Medics 29n; Yorba Linda, CA). The system was calibrated before and during each test by using certificated gas mixtures of known concentrations (4% CO_2_, 16% O_2_ and 80% N_2_; 26% O_2_ and 74% N_2_, SensorMedics). A 3-L syringe (SensorMedics; Yorba Linda, CA) verified the flow of gases. The environmental barometric pressure was measured by a fortin type mercurial barometer (Princo 469; USA), and the temperature and relative humidity by a mason type hygrometer (Taylor 5522S mason hygrometer; Canada). During exercise, expired gases were analyzed with the breath-by-breath system (facemask system). The exercise tests were carried out on an electronic bicycle ergometer (Ergoline 800S, Jaeger; Germany).

#### Graded exercise test

VO_2max_ was calculated from the highest VO_2_ value, attained by each participant during two maximum exercise tests (R^2^ = 0.76, p < 0.001). The initial workload and subsequent increments (in watts) were established 24 h before the test, according to the aerobic capacity of each subject, in such way that the total time of the test ranged between 8 and 12 min. The maximum exercise test was initiated with a workload adjusted between 50 and 75 W, and then a ramp protocol was started with increments of 25–30 W per min in trained participants, and of 15–25 W in untrained participants.

#### Experimental trials

The three different SEFW tests were performed in random order. The watts on bicycle ergometer at each SEFW and for each subject were determined according to his individual workload reached at: a) 1.5 mM below the LT, b) ±1 mM within the LT and c) 2.5 mM above the LT, found in the preceding maximum exercise tests. The individual breaking point on the blood lactate concentration (VO_2_ vs. lactate) was used to determine LT, which was detected before a curvilinear increase in plasma lactate concentration was observed (Beaver et al. 1985). The duration test of the SEFW was 30.0 min, for below LT, and the last 6.3 and 4.0 min, for within LT, and above LT, respectively. In order to minimize the effects of previous exercise tests, subjects were tested every other day. Heart rate was monitored during exercise tests with a telemetric heart rate monitor (Polar F6; Finland).

### Blood lactate assay

Capillary blood samples were taken from the fingertip with a heparinized capillary glass tube. During maximum exercise, blood samples were taken just before starting the test, every 2 min. during the exercise test and at 3 min. post-exercise. During submaximal exercises, blood samples were taken before starting the test, during the first minute, at the middle and just the end of the test. The blood lactate concentrations were determined by using a lactate analyzer (YSI 1500 Sport Lactate Analyzer; OH, U.S.A.).

### Statistical analyses

Results in tables are presented as mean ± standard deviation (SD) and in figures as mean ± standard error (SEM). Differences between groups were determined by unpaired student-t test. The degree of association among variables was assessed by multiple correlation analysis. The independence of associated variables on RER was evaluated with multiple regression analysis and the best model was selected by allpossible regressions procedure. Statistical significance was accepted when α level of p < 0.05. Statistical analyses were conducted with SAS System software, version 8.0.

## Results

**Figure 1.**
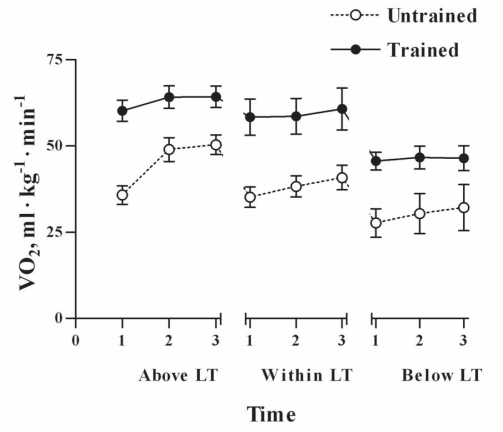
**O_2_ uptake (VO_2_) during submaximal exercises in endurance trained and untrained men.** Open circles, untrained subjects; solid circles, trained subjects. The tests durations were 4.0, 6.3 and 30.0 min for above LT, within LT and below LT, respectively. LT: lactate threshold. VO_2_ values are means of one minute taken at the initiation of exercise (1), at the middle of the test (2) and at the end of the test (3). Data are expressed as mean ± SEM, n = 8.

**Figure 2.**
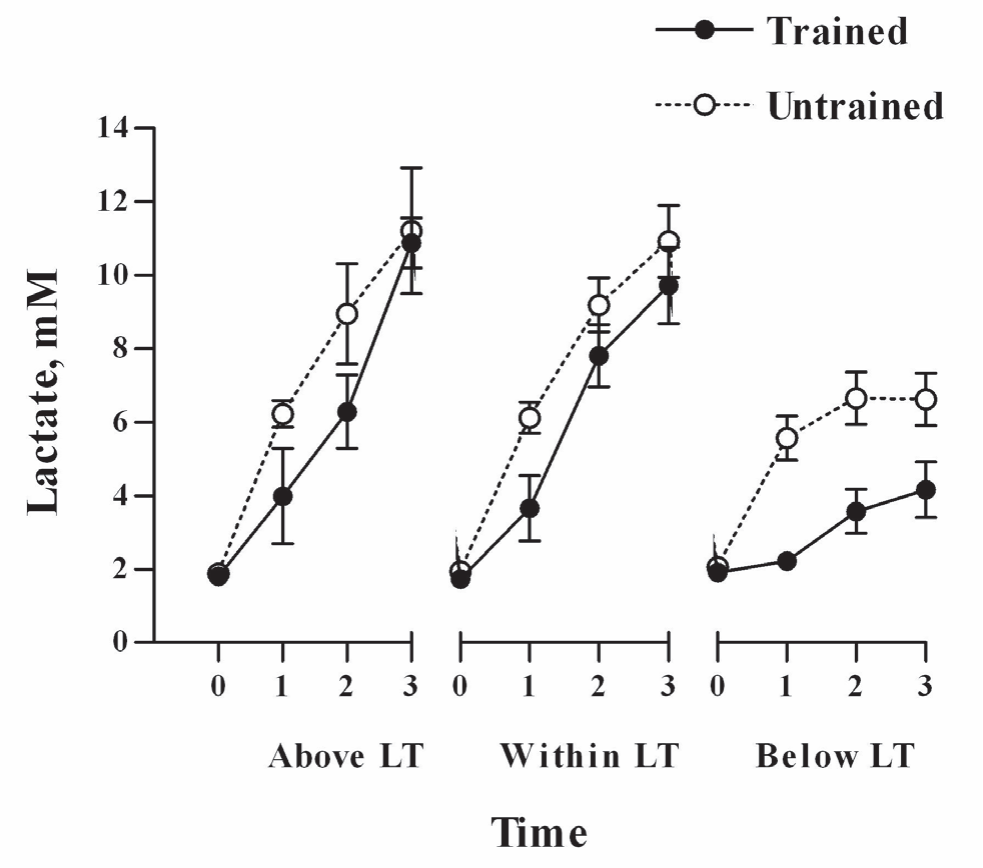
**Blood lactate concentration during submaximal exercises in endurance trained and untrained men.** Open circles, untrained subjects; solid circles, trained subjects. The tests durations were 4.0, 6.3 and 30.0 min for above LT, within LT and below LT, respectively. LT: lactate threshold. Blood lactate was taken at basal (0), the initiation of exercise (1), at the middle of the test (2) and at the end of the test (3). Data are expressed as mean ± SEM, n = 8.

**Figure 3.**
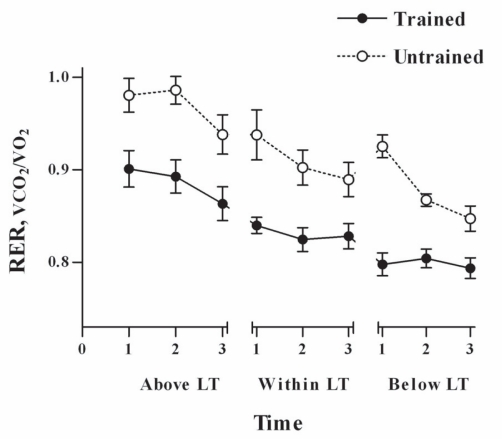
**Respiratory exchange ratio (RER) values during submaximal exercises in endurance trained and untrained men.** Open circles, untrained subjects; solid circles, trained subjects. The tests durations were 4.0, 6.3 and 30.0 min for above LT, within LT and below LT, respectively. LT: lactate threshold. RER values are means of one minute taken at the initiation of exercise (1), at the middle of the test (2) and at the end of the test (3). Data are expressed as mean ± SEM, n = 8.

As expected, all the physical fitness variables: body fat, VO_2max_, workload_max_ and lactate threshold (measured as watts and VO_2_) were significantly different in trained subjects compared to untrained (Table 1). No differences in food intake and percentage of macronutrients composition were observed between the groups (Table 2). Although the trained subjects were working at higher workload intensity (Table 3) and VO_2_ at any SEFW, lower lactate concentrations and lower RER values were observed in them compared to those in untrained subjects (Fig. 1–3).

Considering trained and untrained subjects as a single group, the multiple correlation analyses showed a significant (p < 0.05) association of RER with fitness variables (body fat, maximum heart rate, VO_2max_ and lactate threshold, Table 4) at any SEFW.

Multiple regression analysis was performed to determine any independent association of RER with the aforementioned variables. The results showed that the workload at LT (in watts) was the unique variable for determining changes in RER at below LT (the model explained the variance in 68%). From the same analysis VO_2max_ was the determinant variable for RER at within LT (the model explained the variance in 57%), and together with workload at LT (in watts) were the determinant variables for the changes in RER at above LT (the model explained the variance in 87%) (Table 5).

## Discussion

In the present study, we assessed the RER in trained and physically active untrained fasted men exercising at three different submaximal intensities. Trained subjects, exercising at higher absolute workloads, showed significant lower RER values, higher VO_2_, and lower blood lactate concentrations than untrained subjects. The differences in relative intensity at below LT and within LT may not be statistically significant due to the subject response variability, but the differences are large enough to be physiologically significant. The lower RER values mean that during submaximal exercise, trained subjects oxidized a greater proportion of lipids at higher workload than untrained subjects (Messonnier et al. 2005). Similar differences, at lower intensities, in RER between trained and untrained subjects have been previously found (Bergman and Brooks, 1999). The lower blood lactate concentrations means, that the trained subjects had a more active lipid oxidative metabolism. There was an increase in blood lactate concentration during submaximal exercise, even below LT, in spite of the workout was constant and the VO_2_ did not show any significant change. Our results are in agree with those of Bearden et al. (2004) who find that at two different workouts (40 and 60% of VO_2peak_), lactate increase while the pH decreases. However, increases on blood lactate concentration during exercise could occur without decreases in blood pH (Medbø, 2000). Therefore, increases in blood lactate concentration could be detected without the increase in minute ventilation, induced by the hydrogen ions.

The cross-sectional design of the present study precludes any conclusion regarding the possible mechanisms by which endurance training may lower RER. In longitudinal studies, a decrease in RER has been observed after training at the same absolute workload but not at the same relative intensity (Friedlander et al. 1997; Bergman and Brooks, 1999). Physical exercise increases the activity of some mitochondrial enzymes, like citrate synthase, cytochrome C oxidase and β-hydroxyacyl-CoA dehydrogenase (Tonkonogi et al. 2000; Short et al. 2003; Menshikova et al. 2005). Such biochemical changes drive fatty acid oxidation, which would be reflected as a decrease in RER value. Comparable results have been found in previously untrained subjects after nine consecutive days of endurance training, in whom an increase in both fatty acid translocase (FAT/CD36) and mitochondrial carnitine palmitoyltransferase I (CPT I) gene expressions were observed (Tunstall et al. 2002). Consequently, such enzyme synthesis promoted an increase in fatty acid oxidation and, therefore, a decrease in RER values. On the other hand, physical training also increases the buffer capacity of blood (Juel, 1998) which could contribute to the decrease in the RER values in trained subjects.

The RER could be modified by different factors: food intake (Bergman and Brooks, 1999), type of diet (Spriet and Peters, 1998; Carey et al. 2001), energy balance (Weyer et al. 1999), energy-restricted diet (Keim and Horn, 2004) and extreme ambient temperature (Layden et al. 2002). In our study, the RER evaluations were carried out after 10 to 12-hours of fasting under a controlled temperature, a similar diet and a similar caloric balance; therefore all these variables could not have influenced our results. Both obesity and insulin resistance also modify the RER during exercise (Goodpaster et al. 2002). In our study, only healthy physically active subjects, with low body fat, were included.

The subjects in our study did not work at steady state since the RER, blood lactate, and VO_2_ values were changing throughout any SEFW. Similar results on RER have been found (Bergman and Brooks, 1999; Campbell et al. 2004), where the steady state is not reached despite the subjects work at constant exercise intensity. The RER decreases at the end of the exercise are indicative of both, a shift towards lipid metabolism (Bier and Young, 1983) and a lower compensatory hyperventilation to lactic acidosis.

Multiple correlation analysis showed a systematic association between RER at any SEFW and physical fitness variables (body fat, maximum heart rate, VO_2max_ and lactate threshold). Furthermore, results of multiple regression analysis showed that the aerobic fitness indicators VO_2max_ and lactate threshold determine between 57% and 87% of RER variance during exercise. The association of the RER with variables of physical fitness, like anthropometric characteristics, VO_2max_ and lactate threshold, has not been clearly established. Goedecke et al. (Goedecke et al. 2000), studying the RER at rest and during three different sub maximum exercises at relative intensities, did not find a correlation between RER and either body fat or VO_2max_. Nevertheless, through a multivariate analysis, they determined that the training volume is responsible for the changes on RER values at rest and during exercise. In addition, these authors found that citrate synthase activity, an indicator of aerobic capacity, had an influence on the RER values during exercise. Based on these data, Goedecke et al. (Goedecke et al. 2000), established the associations between variables of physical fitness and RER. The present work strengthens these results. On the other hand, while the untrained subjects did not expend energy in previous exercises, the trained subjects expended 4704 ± 2323 kJ (per session), as calculated by training volume. Since, as reported by Goedecke et al. (Goedecke et al. 2000) training volume is an important determinant of both resting and sub maximum exercise RER, this difference probably influenced our results. In addition, acute exercise affects lipid oxidation (Jamurtas et al. 2004), hence we attempted to minimize this effect by determining the RER, after a period of at least 24 h without any physical exercise.

Maximal incremental exercise testing is the gold standard in the cardio-respiratory fitness assessment and provides a wealth of clinically diagnostic and prognostic information (Whaley, 2006); however, not all people can do it (Vanhees et al. 2005; D’Alonzo et al. 2006). The results of the present work suggest the possible application of RER during submaximal test at below LT intensities, and being an useful metabolic fitness indicator in special populations and subjects with reduced exercise tolerance. For that reason, the LT or VO_2max_ should not be necessary because, as independent parameter is only necessary work at one submaximal and confortable intensity. However, because of we have not tested a specific population in this study; this proposal should be taken with caution but deserves future validation. For the above mentioned, since the accurate quantification of physical activity becomes essential in determining the health status, and in evaluating the intervention programs effectiveness (Whaley, 2006), the evaluation of health-related physical fitness, besides of body composition, muscular strength, flexibility, and cardio-respiratory measurements, has to include the total oxidative metabolism assessment measured as RER.

## Conclusions

Trained subjects showed higher oxidative metabolism and lower RER values than the untrained subjects, even when exercising at similar relative submaximal workloads. The physical fitness indicators (body fat, heart rate, VO_2max_, and lactate threshold, measured as VO_2_ and watts) were associated with RER in both groups despite of training condition; in addition, the aerobic fitness (VO_2max_ and lactate threshold) determine more than 57% of RER variance during exercise.

Since RER provides information about physical fitness, RER measurement could be considered in the evaluation of physical activity intervention programs; especially in people with a limited capacity for physical activity, because its assessment applied under submaximal exercise conditions and at low intensity is health, safety, easy and reliable.

## Practical Implications

- The RER could be considered as an additional fitness indicator at low-moderate intensity and being independent of LT and VO_2max_.
- At similar relative submaximal exercise intensities, endurance trained subjects oxidized a greater proportion of lipids than untrained male.
- In physically active healthy men, the physical fitness parameters are strongly associated with the RER during exercise.

## Lineaments for Future Investigations

The main obstacle of this study was to maintain working all our subjects more than 4 min above of the LT. For this reason, the high-work intensity has not to be far away the LT.

To realize more research looking for associations of RER with other fitness indicators and heath parameters in general and special populations.

## Figures and Tables

**Table 1.** Physical and physiological characteristics^1^ and RER at rest of untrained compared to trained men.

	**Untrained (n = 8)**	**Trained (n = 8)**
Age, yr	21.9 ± 2.8	22.9 ± 4.5
Body mass, kg	72.2 ± 7.7	67.1 ± 5.4
Height, cm	176.6 ± 5.4	172.7 ± 4.0
Body fat, %	16.6 ± 3.8	10.6 ± 2.4**
HR_max_, beats • min^−1^	199 ± 5	189 ± 7*
VO_2max_, ml • kg^−1^ • min^−1^	51.6 ± 5.8	68.9 ± 6.3**
Workload_max_, watts	254 ± 46	352 ± 30**
Lactate at breaking point, mM	3.6 ± 0.2	3.5 ± 0.3
Lactate_max_, mM	10.0 ± 3.7	11.6 ± 2.3
Workload at LT, watts	162 ± 32	256 ± 29***
VO_2_ at LT, ml • kg^−1^ • min^−1^	31.8 ± 6.6	53.2 ± 10.5***
RER at rest, VCO_2_ • VO_2_^−1^	0.79 ± 0.04	0.82 ± 0.05

Values are expressed as mean ± SD.

1 during maximal exercise test. HR_max:_ maximal heart rate; LT: lactate threshold; VO_2max_: maximal O_2_ uptake; max: maximal; RER: Respiratory exchange ratio. Unpaired student-*t* test.

* p < 0.05,

** p < 0.01 and

*** p < 0.001 vs. untrained.

**Table 2.** Food intake^1^.

	**Untrained**	**Trained**
Caloric intake, kcal • day^−1^	2341 ± 451	3009 ± 879
Carbohydrates, % caloric	50.1 ± 8.0	51.2 ± 7.5
Lipids, % caloric	31.7 ± 5.5	30.7 ± 5.2
Proteins, % caloric	18.2 ± 3.6	18.1 ± 3.1

Values are expressed as mean ± SD.

1 Average recalls of 3 sequential days of diet.

**Table 3.** Workload during exercise at three different intensities.

	**Workload intensity (% of maximal^1^)**		**Workload intensity (watts)**	
SEFW	Untrained	Trained	Untrained	Trained
Below LT	52.2 ± 10.0	57.6 ± 4.5	135 ± 39	216 ± 57*
Within LT	65.9 ± 14.8	75.5 ± 4.1	164 ± 35	260 ± 30**
Above LT	90.8 ± 37.0	87.2 ± 4.7	238 ± 41	300 ± 9**

Values are expressed as mean ± SD. LT: lactate threshold; SEFW: submaximal exercise at fixed workload.

1 % of maximal watts reached during maximal test. Unpaired student-*t* test.

* p < 0.05,

** p < 0.01 vs. untrained.

**Table 4.** Correlation matrix for fasting RER during exercise at different fixed workload intensities on both groups.

**RER at**	**% of body fat**	**HR**_max_	**VO**_2max_	**VO**_2_**at LT**	**Workload at LT**
Below LT	0.61*	0.54§	−0.71*	−0.72**	−0.83**
Within LT	0.54*	0.55*	−0.75**	−0.62*	−0.71**
Above LT	0.67*	0.68*	−0.89***	−0.85***	−0.87***

Trained and untrained groups were analyzed as a single group. HR_max_: maximal heart rate; LT: lactate threshold; RER: respiratory exchange ratio; VO_2max_: maximal O_2_ uptake. Multiple correlation analysis.

§ P = 0.05,

* p < 0.05,

** p < 0.01,

*** p < 0.001.

**Table 5.** Multivariate analysis for fasting RER during exercise at different fixed workload intensities on both groups.

**RER at**	**Equation**	**R**^2^	**p level**
Below LT	= 0.995 – 0.0007 workload at LT	0.68	0.001
Within LT	= 1.105 – 0.0039 VO_2max_	0.57	0.001
Above LT	= 1.225 – 0.0004 workload at LT – 0.0032 VO_2max_	0.87	0.048

LT: lactate threshold; RER: respiratory exchange ratio; VO_2max_: maximal O_2_ uptake. Multiple regression analysis.
